# miRNA-Directed Regulation of the Main Signaling Pathways in Thyroid Cancer

**DOI:** 10.3389/fendo.2019.00430

**Published:** 2019-07-02

**Authors:** Julia Ramírez-Moya, Pilar Santisteban

**Affiliations:** ^1^Instituto de Investigaciones Biomédicas “Alberto Sols”, Consejo Superior Investigaciones Científicas and Universidad Autónoma de Madrid (CSIC-UAM), Madrid, Spain; ^2^Centro de Investigación Biomédica en Red de Cáncer (CIBERONC), Instituto de Salud Carlos III (ISCIII), Madrid, Spain

**Keywords:** miRNAs, MAPK, PI3K, TGFβ, thyroid, cancer

## Abstract

In the last two decades, great strides have been made in the study of microRNAs in development and in diseases such as cancer, as reflected in the exponential increase in the number of reviews on this topic including those on undifferentiated and well-differentiated thyroid cancer. Nevertheless, few reviews have focused on understanding the functional significance of the most up- or down-regulated miRNAs in thyroid cancer for the main signaling pathways hyperactivated in this tumor type. The aim of this review is to discuss the major miRNAs targeting proteins of the MAPK, PI3K, and TGFβ pathways, to define their mechanisms of action through the 3′UTR regions of their target genes, and to describe how they affect thyroid tumorigenesis through their actions on cell proliferation, migration, and invasion. Given the importance of miRNAs in cancer as diagnostic, prognostic and therapeutic candidates, a better understanding of this cross-talk might shed new light on the biomedical treatment of thyroid cancer.

## Introduction

A wealth of studies over the last decade has highlighted the importance of microRNAs (miRNAs) in cancer development. This is likely due to the extreme versatility of these small molecules as key regulators of gene expression. miRNAs are defined as small non-coding RNAs of length 19–25 nucleotides that control gene expression at the posttranscriptional level by hybridizing to target mRNAs at the 3′ untranslated region (3′UTR), suppressing their translation or inducing their degradation.

The finding that one miRNA can target a large number of different mRNAs, and that one mRNA molecule can be targeted by numerous miRNAs, gives these molecules the potential to fine-tune gene expression levels in physiological and pathological processes such as cancer (1–3). Indeed, many miRNAs are known to target mRNAs involved in cancer including oncogenes and tumor suppressors, and therefore play essential roles in cancer initiation, progression and metastasis formation (4). Conversely, it is also recognized that oncogenes and tumor suppressors exert their actions by controlling the expression of specific miRNAs. These finding overall support the existence of feedback mechanisms between miRNAs and their targets in human cancers including thyroid cancer, which is the most commonly occurring endocrine malignancy and whose incidence has increased steadily over the last four decades (5, 6).

Thyroid cancer of epithelial/follicular origin comprises different histological subtypes. Carcinomas with the ability to maintain differentiation are termed well-differentiated thyroid carcinomas (WDTC) and include two subtypes—papillary (PTC) and follicular (FTC) thyroid carcinoma. The other subtypes are poorly differentiated (PDTC) and (undifferentiated) anaplastic (ATC) thyroid carcinomas (7). PTCs are the most frequent differentiated thyroid carcinomas and represent 80–85% of all thyroid malignancies, whereas ATC is the less frequent malignancy but the most aggressive, and has an extremely poor prognosis (median survival of 6 months after diagnosis).

It is well-accepted that thyroid cancer fits a stepwise model of progression, which considers thyroid carcinomas as tumors accumulating mutations that drive tumorigenesis through a dedifferentiation process. Analysis of the genome sequence has recently revealed the genomic landscape of these tumors (8, 9), which has led to a redesign of the stepwise model of thyroid cancer progression. Depending on the key oncogenic drivers associated with PTC and FTC, tumors are grouped together into BRAF-like and RAS-like, as a function of their characteristic molecular features. Typically, PTCs are considered BRAF-like tumors, whereas FTCs are considered RAS-like. However, the follicular variants of PTC are considered RAS-like, and consequently more akin to FTC. Additional drivers are involved in the progression to PDTC and to ATC [reviewed in (10)].

The molecular classification of thyroid carcinomas is based on the mutations in the main known signaling pathways. In the case of PTC, it is accepted that it is a MAPK pathway-driven cancer with RAS and BRAF activating mutations being the main oncogenic players, which are found in an exclusive manner (11). The follicular variant of PTC, a RAS pathway-like tumor, shares characteristics with PTC and FTC and is associated with the activation of both MAPK and PI3K signaling (12). In the case of FTC, the main driver is RAS. While RAS proteins can activate both the MAPK and PI3K pathways, the oncogenic variants of RAS are likely more dependent on PI3K activation to initiate tumorigenesis (13). Finally, mutations activating both MAPK and PI3K have been detected in PDTC and ATC, which together with mutations in genes involved in aggressiveness increase the mutational burden in these tumors (13).

Considering thyroid cancer, one of the most important consequences of the new era of genomic studies is the possibility to differentiate between the two genetic types, particularly the manner in which BRAF and RAS signaling promotes tumor development and growth. BRAF-driven tumors have high MEK-ERK activity whereas RAS-driven tumors show the opposite and activate PI3K. BRAF-driven tumors are very heterogeneous in terms of gene expression, miRNA profiles, and epigenetic alterations. TGFβ signaling contributes to this heterogeneity, as BRAF induces the secretion of functional TGFβ, which together with MAPK signaling supports cell migration, invasion and epithelial-mesenchymal transition (EMT) (14). Accordingly, PTC tumors express high levels of TGFβ and other components of this pathway such as its receptor TβRII and phosphorylated SMAD2 protein (14–16). These data indicate that TGFβ/SMAD activity is associated with PTC invasion, nodal metastasis and BRAF status, and should be considered an important pathway in thyroid cancer.

Regarding miRNAs, which are the focus of this review, The Cancer Genome Atlas (TCGA) research network recently revealed that activation of the aforementioned oncogenes deregulates miRNA expression in thyroid cells (8), suggesting that miRNA expression patterns are relevant for pathogenesis in patients, as they contribute to loss of differentiation and induce tumor progression. The known mechanisms of miRNA deregulation in cancer include amplification, deletion, mutation, and epigenetic silencing (17, 18). As an illustration of the complexity of these regulatory networks, TCGA analysis revealed the presence of six miRNA clusters in PTC. Cluster 1 is associated with RAS-mutated tumors and the follicular variant and it is enriched with the miRNAs miR-181 and miR-182, whereas the BRAF tumors are defined by five miRNA clusters (Clusters 2–6). Cluster 5 (enriched with miR-146b and miR-375, and low levels of miR-204), together with Cluster 6 (with high levels of miR-21 and low levels of miR-204), are associated with the less-differentiated tumors and a higher risk of recurrence (10). Beyond these clinically relevant miRNA expression patterns, the role(s) of specific miRNAs in thyroid carcinogenesis is under intensive investigation.

Individual miRNAs regulate important processes of tumor biology and new examples of miRNAs that coordinately regulate cancer pathways are reported almost daily. In the context of thyroid cancer, miRNA regulation by the major signaling pathways has been reviewed in depth; however, to our knowledge, a comprehensive analysis of the most important miRNAs that control the main signaling pathways activated in thyroid cancer—MAPK, PI3K, and TGFβ–has received less attention. Thus, we will review here what we know about miRNA regulation of the main signaling pathways altered in thyroid cancer. To this end, we will focus on the most important miRNAs up- and down- regulated in thyroid cancer and how they target components of the signal transduction pathways involved in the MAPK, PI3K, and TGFβ pathways.

Our approach will be an analysis of the signaling pathways from the membrane to the nucleus, describing both up- and down-regulated miRNAs in thyroid cancer that regulate the main proteins involved in these pathways.

### MAPK Signaling Pathway

This MAPK signaling pathway employs a series of protein kinases to transmit signals from the cell membrane to the nucleus, in order to control several cellular processes such as proliferation, differentiation, migration, invasion and apoptosis. MAPK hyperactivation has been implicated in many different pathologies including cancer (19).

Mechanistically, MAPK signaling functions through growth factor and mitogen binding to plasma membrane tyrosine kinase receptors, inducing receptor dimerization and autophosphorylation of specific residues that are recognized by the adaptor proteins SHC1 and GRB2. In turn, these adaptor proteins recruit GTPase exchange factors such as SOS to promote GDP–GTP exchange on RAS proteins. After their activation, RAS proteins recruit to the plasma membrane RAF (BRAF or RAF1), inducing the sequential activation of MEK and ERK. This process is enabled by scaffold proteins that modulate pathway activation, being its components translocate to various cellular compartments, particularly the nucleus where proteins involved in proliferation such c-FOS, c-JUN, c-MYC, or ELK1 are activated (13).

#### Upregulated miRNAs Modulating the MAPK Pathway Activity in Thyroid Cancer

Few studies have described upregulated miRNAs targeting the MAPK pathway in thyroid tumors. Because MAPK signaling must be activated to induce tumorigenesis, miRNAs that are upregulated could inhibit antagonists of this pathway, which would maintain the hyperactivation observed in thyroid cancer. Indeed, it has been established that miR-21 targets inhibitors of the RAS-MAPK pathway (*SPRY1, SPRY2, BTG2*, and *PDCD4*), therefore activating this pathway in lung cancer (20). miR-21 is a key oncogenic miRNA upregulated in many cancers, including PTC, but further studies will confirm the role of inhibit these targets in thyroid tumors. Interestingly, mutated *BRAF* might regulate or interact with miRNAs in the pathogenesis and progression of PTC, as the expression levels of several important upregulated miRNAs such as miR-221, miRNA-222, miRNA-146b, and miRNA-181, were shown to be significantly higher in PTC patients with BRAF mutations (21, 22).

#### Downregulated miRNAs Modulating the MAPK Pathway in Thyroid Cancer

The lethal miRNA family let-7, which was first discovered in the worm (23), was also the first to be implicated in human disease through their regulation of the RAS-ERK/MAPK pathway (24). Indeed, the 3′ UTR of all three RAS genes (*HRAS, KRAS*, and *NRAS*) encloses multiple binding sites for let-7 family members, and enforced expression of let-7 in human cancer cells reduces RAS protein levels. Accordingly, the downregulation of let-7 expression reported in several human cancers could lead to RAS pathway activation. This mechanism has also been observed in thyroid cancer cells (25), where RAS activation and downregulation of some members of the let-7 family was noted. The sweeping importance of let-7 downregulation in cancer is supported by the finding that let-7 can suppress tumor growth in several cancers such as lung or colon cancer (26).

Regarding thyroid carcinomas, three independent studies have shown that let-7f is downregulated in PTC (27–29), although the analysis of TCGA data would indicate that it is not significantly downregulated. Let-7f has been linked to RAS protein levels in PTC (30). Moreover, stable transfection of let-7f in TPC-1 cells, a human PTC cell line that spontaneously harbors the *RET/PTC1* oncogene, inhibits MAPK activation, and leads to an obvious reduction in cell proliferation and the induction of thyroid differentiation markers (25).

Epidermal growth factor receptor (EGFR), a tyrosine kinase receptor that can shuttle from the membrane to the nucleus, is an oncogene overexpressed in a variety of human cancers including thyroid cancer (31–34), and mediates cell proliferation via ERK and AKT signaling. Basing their study on previous works showing a general downregulation of miR-137 in many different types of human cancer, Luo et al. (33) used real-time PCR to show that mean expression of miR-137 was lower in samples of fresh PTC tissue than in adjacent normal tissue (*n* = 25), although according to TCGA data miR-137 expression is not significantly different (8). Interestingly, the authors found that miR-137 directly targets EGFR and used loss- and gain-of-function studies to show that an miR-137 mimic significantly downregulated EGFR mRNA and protein, whereas an miR-137 inhibitor had the opposite effect. miR-137 was also found to downregulate cell proliferation, colony formation ability, and invasion, and negatively regulated ERK and AKT signaling. Interestingly, EGFR depletion abrogated the effect of miR-137 inhibition on ERK and AKT signaling, suggesting that the role of miR-137 on ERK and AKT signaling was EGFR dependent (33). The EGFR family member, ERBB2 (encoding the neuregulin receptor), is also considered an oncogene and is overexpressed in many cancers (35, 36). ERBB2 is associated with RAS-MAPK and PI3K-AKT signaling and its overexpression would likely reduce the sensitivity of cells to chemotherapy and radiotherapy (36, 37). ERBB2 was recently described to be targeted by miR-375 in PTC tissue (37). Interestingly, whereas miR-375 is significantly upregulated in PTC according to both TCGA data (8) and an independent miRNA deep sequencing study (38), Wang et al. (37) reported miR-375 as being downregulated in PTC tissues (*n* = 60, real-time PCR assay) and cell lines. The authors studied the biological effect of miR-375 on human PTC cell lines establishing that its overexpression inhibited proliferation and induced apoptosis *in vitro*, and decreased migration and invasion *in vivo*. Nevertheless, the precise mechanism of action miR-375 and whether it is associated with MAPK/ERK or PI3K/AKT pathways. was not addressed, and thus further studies are needed to better understand the mechanism of action of miR-375 in thyroid cancer.

Other studies have described several downregulated miRNAs that inhibit the MAPK/ERK pathway downstream of its receptors. For instance, Liu et al. identified miR-4728 as a significantly downregulated miRNA in 18 pairs of human PTC and non-cancerous normal tissue, as determined by real-time PCR analysis (39). However, no changes were observed in TCGA data (8). Functionally, miR-4728 inhibits PTC cell proliferation and decreases the mRNA and protein levels of the GTPase exchange factor SOS1, thereby inhibiting MAPK/ERK activity (39). Similarly, miR-20b has also been described to directly target *SOS1*, in addition to the extracellular signal-regulated kinase 2 (*ERK2*), consequently downregulating the signaling pathway (40). Depletion of *SOS1* or *ERK2* has similar effects to that observed from miR-20b overexpression, decreasing cell viability and invasion, and the rescue of these two genes partially reversed the effects of miR-20b in a PTC cell line. Finally, overexpression of miR-20b decreased tumor growth in a xenograft model, leading the authors to conclude that miR-20b has a tumor suppressor function *in vivo* (40). In contrast to TCGA data (8), which did not find this miRNA to be significantly differentially expressed, Hong et al. found miR-20b downregulated when analyzing 47 pairs of PTC and their matched adjacent normal tissue (40). This result was consistent with an earlier study demonstrating that miR-20b is significantly downregulated in PTC by using next-generation deep sequencing (*n* = 14) and microarray analysis (*n* = 9) (41).

**Figure 1:**
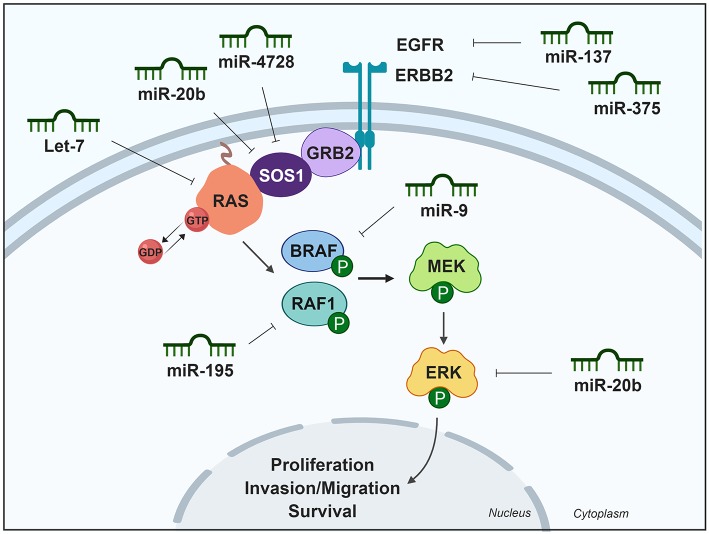
Differentially expressed miRNAs in thyroid cancer tissues modulating the MAPK pathway. Green miRNAs represent downregulated miRNAs when comparing tumoral vs. control tissue. Created with BioRender.

The RAF kinase family comprises three serine/threonine-specific protein kinase isoforms: A-RAF, B-RAF, and RAF1. RAF1 is directly downstream of and can be activated by RAS. Once activated, cellular RAF1 guides the receptor signals from the cell membrane to the nucleus by phosphorylating and activating the dual specificity protein kinases MEK1 and MEK2, therefore activating the MAPK/ERK pathway and regulating cell cycle, proliferation, apoptosis, and migration. RAF1 is upregulated in thyroid cancer, which could be in part caused by the deregulation of miR-195 (42). This miRNA is downregulated in thyroid cancer (8, 42) and directly targets *RAF1* to block thyroid cancer cell proliferation. Another member of this kinase family, BRAF, is also an effector of RAS and a main component of the MAPK/ERK pathway. Mutations in *BRAF* induce uncontrolled and persistent activation of this kinase-signaling pathway, causing over-proliferation of cancer cells. BRAF expression is controlled by miR-9, which is downregulated in PTC according to both TCGA data and to a study by Guo et al. (8, 43). These authors found that miR-9 directly targets the BRAF 3′UTR. Furthermore, miR-9 suppress the viability of PTC cells by inducing apoptosis, therefore acting as a tumor suppressor miRNA (43). A summary of miRNAs and their targets in the MAPK pathway is shown in Table 1 and is schematically represented in Figure 1.

**Table 1:** Summary of miRNAs affecting the main signaling pathways in thyroid cancer.

**miRNA**	**Target/s**	**Pathway affected**
**UPREGULATED miRNAs**		
miR-146b	PTEN	PI3K
	ST8SIA4	PI3K
	SMAD4	TGFβ
miR-21	PTEN	PI3K
miR-146a	ST8SIA4	PI3K
miR-34a	GAS1	PI3K
miR-221	p27^Kip1^	PI3K
miR-222	p27^Kip1^	PI3K
miR-29b	SMAD3	TGFβ
miR-23b	SMAD3	TGFβ
**DOWNREGULATED miRNAs**		
Let-7	RAS	MAPK
		PI3K
miR-137	EGFR	MAPK
		PI3K
miR-375	ERBB2	MAPK
		PI3K
miR-4728	SOS1	MAPK
miR-20b	SOS1	MAPK
	ERK2	MAPK
miR-195	RAF1	MAPK
miR-9	BRAF	MAPK
miR-126	PIK3R2	PI3K
miR-451a	MIF	PI3K
	AKT1	PI3K
	c-MYC	PI3K
miR-145	AKT3	PI3K
miR-99a	mTOR	PI3K
miR-663	TGFβ1	TGFβ
miR-7	TGFβRII	TGFβ
miR-144	TGFβRII	TGFβ
miR-200	TGFβRI	TGFβ
	SMAD2	TGFβ
miR-30	SMAD2	TGFβ

### PI3K Signaling Pathway

The phosphoinositide 3-kinase–protein kinase B/AKT (PI3K-PKB/AKT) pathway a critical molecular signaling pathways involved in key cellular processes. Protein kinase B (also known as AKT) is the main downstream effector of PI3K, and its activation regulates several cellular processes, such as proliferation, growth, apoptosis, cell survival and angiogenesis in several cell systems, including thyroid cells. Constitutive or enhanced signaling of this pathway is common in thyroid cancer (44, 45), and elevated AKT activity has been associated with tumor size and invasion in both FTC and PTC, with the exception of tumors with BRAF activating mutations (44). Mutations in different components of the PI3K pathway are rare in WDTC, but have a significant prevalence in PDTC and ATC, suggesting a role for PI3K activation in the progression to undifferentiated carcinomas (13).

#### Upregulated miRNAs Modulating the PI3K Pathway Activity in Thyroid Cancer

The tumor suppressor PTEN is the main negative regulator of the PI3K pathway. PTEN antagonizes the PI3K pathway via its lipid phosphate activity, which diminishes the cytosolic localization of AKT and its subsequent activation. Some upregulated miRNAs have been shown to inhibit PTEN expression, thereby increasing PI3K pathway activity. For example, it was recently shown that miR-146b is the most upregulated miRNA in thyroid cancer (8, 38) and PTEN is one of its main targets. In a thyroid cell system, miR-146b expression decreased the expression of PTEN, which was accompanied by PI3K/AKT hyperactivation, leading to the nuclear exclusion of two main downstream effectors, FOXO1 and p27^Kip1^ and a corresponding increase in cellular proliferation. Moreover, miR-146b-induced repression of PTEN protected cells from apoptosis and increased migration and invasion, chiefly by regulating genes implicated in EMT (46). Interestingly, intratumor administration of a miR-146b inhibitor (antagomiR-146b) blocked tumor growth *in vivo* in a xenograft mouse model (46), and a similar result was obtained in a thyroid orthotopic tumor model after systemic administration of the antagomiR (47). Importantly, this inhibition increased the protein levels of the miR-146b target PTEN (46). In addition to miR-146b, miR-21 is a highly relevant tumor-associated miRNA and is the most frequent upregulated oncomiR in solid tumors, including PTC. miR-21 is regulated by the oncoprotein RAS through activator protein-1 (AP-1) (48). PTEN has also been identified as a target of miR-21 (49), and this was subsequently experimentally validated in a thyroid cell system (48). Consequently, PTEN expression is repressed by AP-1 in response to RAS signaling, which is mediated by miR-21. Further, the same group described that the induction of miR-21 requires the activation of at least two RAS downstream pathways, MAPK and PI3K itself (50).

The PI3K/AKT signaling pathway has been reported to be under regulation by the sialyltransferase family in cancer (51). miR-146a is also upregulated in thyroid carcinoma (8, 38) and bioinformatic analyses showed that miR-146a, together with miR-146b, target the sialyltransferase family member ST8SIA4. *In vitro* analysis showed that ST8SIA4 transfection decreased the invasiveness of an miR-146a/b-overexpressing FTC cell line, whereas ST8SIA4 silencing inverted the effects of miR-146a/b inhibition. PI3K p110α, phosphorylated AKT and phosphorylated mTOR were all increased in miR-146a/b-overexpressing cells and their expression was partially reduced after ST8SIA4 restoration. Overall, these results suggest that miR-146a/b activates the PI3K-AKT-mTOR signaling pathway, which is suppressed by ST8SIA4 (52).

Other upregulated miRNAs have been found to play activating roles in the PI3K/AKT signaling pathway. miR-34a, which is upregulated in thyroid cancer (8, 38, 53), promotes cell proliferation and colony formation and inhibits apoptosis. Silencing of *GAS1* had similar effects, in terms of cell growth, as the overexpression of miR-34a. Furthermore, PI3K is activated in PTC cells that overexpress the miR-34a, and depletion of AKT reversed the cell growth, and the anti-apoptotic effects of miR-34a. Thus, miR-34a is thought to function via activation of the PI3K pathway, likely by repressing *GAS1* expression and thereby activating RET and downstream PI3K pathways (53). By contrast, Liu et al. (54) described miR-34a as a downregulated tumor-suppressor miRNA in thyroid cancer tissue and cell lines. They showed that miR-34a decreases the phosphorylation of AKT via MET inhibition. MET acts as a receptor tyrosine kinase and plays key roles in promoting cell growth and proliferation by transducing extracellular stimuli to intracellular signaling circuits including the PI3K pathway.

Activated AKT phosphorylates several targets, such as p27^Kip1^, a kinase inhibitor protein that regulates the cell cycle by preventing the transition from G1 to S phase. Phosphorylation of p27^Kip1^ impairs its nuclear import and leads to cytoplasmic accumulation and cell resistance to G1 arrest (55). Some upregulated miRNAs have been shown to target this effector directly, including miR-221 and miR-222, which negatively regulated p27^Kip1^ expression through two target sites in its 3′UTR region. Consistently, enforced expression of miR-221 and miR-222 induced a PTC cell line to progress to the S phase of the cell cycle (28). Moreover, an inverse correlation was found between miR-221 and miR-222 up-regulation and down-regulation of p27^Kip1^ protein levels in human thyroid papillary carcinoma (28).

#### Downregulated miRNAs Modulating the PI3K Pathway in Thyroid Cancer

The PI3K pathway can also be regulated by miRNAs that are under-expressed in thyroid cancer, usually by inhibiting genes and regulators of this pathway and thereby decreasing pathway activity. As mentioned previously, inhibition of the EGFR and ERBB2 receptors by miR-137 and miR-375, respectively, does not only affect the MAPK pathway, but also the PI3K pathway. Class I PI3Ks are heterodimeric molecules composed of a p110 catalytic subunit and a p85 regulatory subunit (45). miR-126 was found to act as a proliferation suppressor in BRAF-mutated undifferentiated thyroid cancer cell lines by targeting *PIK3R2* encoding subunit p85β, and reducing protein translation, as demonstrated by miR-126 mimic transfection and reduction of p85β and pAKT protein levels (56).

miR-451a is one of the most downregulated miRNAs in thyroid cancer, and reduces the protein levels of its recognized targets *MIF, AKT1*, and *c-MYC* in PTC cell lines, attenuating AKT pathway activation (57). The pro-inflamatory cytokine MIF (Macrophage migration inhibitory factor) is overexpressed in various tumors, where it promotes tumor growth by the stimulation of multiple signaling cascades, including the AKT pathway (58). Finally, c-MYC is a well-studied oncogenic transcription factor that integrates signals from multiple pathways, including the AKT pathway. c-MYC regulates gene expression and induces cell proliferation, differentiation, and transformation (57, 59). Overall, it seems that miR-451a displays tumor suppressor functions by targeting multiple elements of the PI3K/AKT pathway in PTC.

miR-145 is also downregulated in thyroid cancer (8, 60), and directly targets *AKT3*, reducing AKT levels and inhibiting the PI3K pathway (60). Overexpression of miR-145 in thyroid cancer cell lines resulted in decreased cell proliferation, migration, invasion, VEGF secretion and E-cadherin expression, and decreased tumor growth and metastasis in a xenograft mouse model. Therefore, miR-145 mediates its effects through the PI3K/AKT pathway, and could be an important regulator of thyroid cancer growth.

**Figure 2:**
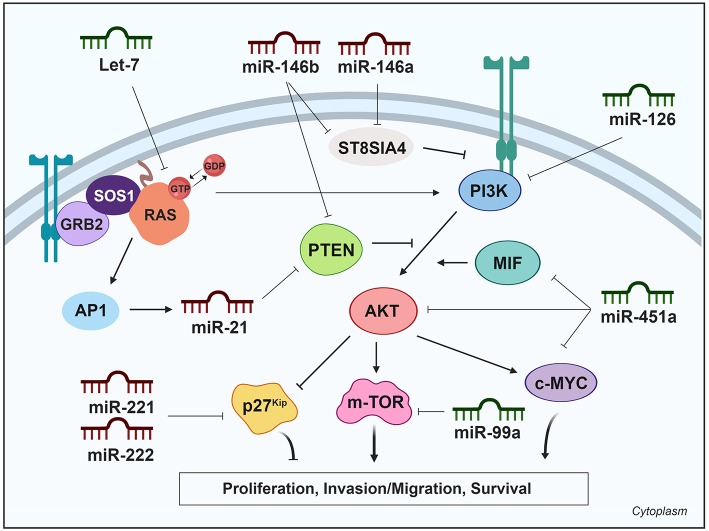
Differentially expressed miRNAs in thyroid cancer tissues modulating the PI3K pathway. Green miRNAs represent downregulated miRNAs when comparing tumoral vs. control tissue and red miRNAs represent the upregulated. Created with BioRender.

A major response of the PI3K pathway is the activation of mTOR, which is enhanced by AKT. mTOR is a serine/threonine protein kinase in the PI3K signaling cascade (61). mTOR phosphorylates and activates p70 S6 kinase, and also inhibits eukaryotic translation initiation factor 4E binding protein, resulting in enhanced protein synthesis and cell proliferation. Yang et al. were the first to describe that miR-99a directly targets the mTOR signaling pathway in breast cancer side population cells (62). Subsequently, miR-99a was identified as a downregulated miRNA in PTC (8) and ATC (63) samples. It was experimentally confirmed that miR-99a reduces tumorigenicity in anaplastic thyroid cells *in vitro* and *in vivo* by targeting and reducing mTOR levels, and decreasing downstream phosphorylated proteins eukaryotic translation initiation factor 4E binding protein and p70 S6 kinase (63). A summary of miRNAs and their targets in the PI3K pathway is shown in Table 1 and is schematically represented in Figure 2.

### TGFβ Signaling Pathway

The consequence of activated TGFβ signaling is context-dependent and this factor can be classified both as a tumor suppressor and a tumor promoter. Thyroid cancer is an example of this paradox; however, the mechanism underlying this switch of TGFβ action is not well-understood. In thyroid tumors, TGFβ commonly acts as a tumor suppressor at early stages but as a tumor promoter at later stages. The important role of TGFβ activation in BRAF-mutated papillary thyroid tumors has been well-described (14). In early stages of tumorigenesis, the TGFβ pathway is a negative regulator of thyroid follicular cell growth, but the mechanism by which thyroid cancer cells evade its inhibitory signal remains unclear. Some upregulated miRNAs target components of this pathway to inhibit the anti-proliferative signals. By contrast, at later states, the TGFβ pathway induces EMT, migration and invasion, cooperating with BRAF (14).

TGFβ1 is a member of a family of cytokines involved in cell growth (64), differentiation, and apoptosis. This cytokine binds to the type II TGFβ receptor (TGFβRII), recruiting type I TGFβ receptor (TGFβRI) phosphorylating it, initiating signal transduction mediated by downstream SMAD proteins (65, 66). The activated receptor phosphorylates the cytoplasmic transcription factors SMAD2 and SMAD3 (SMAD2/3), allowing them to translocate to the nucleus. SMAD4 acts as a partner with SMAD2/3 to facilitate this process. This translocated heteromeric complex then controls the gene expression contributing to thyroid cancer.

#### Upregulated miRNA Modulating the TGFβ Pathway Activity in Thyroid Cancer

Several miRNAs are linked to the TGFβ signaling pathway by targeting some of its components. For example, miR-23b and miR-29b are upregulated in PTC according to TCGA data (8) and it has been described that they are regulated by TSH, and their upregulation is required for thyroid cell growth (67). Both miRNAs target SMAD3, inhibiting the TGFβ pathway and promoting thyroid cell growth. Interestingly, an increased expression of these miRNAs was also detected in experimental and human goiter—an enlargement of the thyroid gland (67). In addition, miR-146b is, as previously mentioned, the most upregulated miRNA in PTC and is regarded as an important diagnostic marker. miR-146b binds directly to the 3′UTR of SMAD4. The inhibition of miR-146b was found to increase SMAD4 levels in PTC cell lines, consequently increasing the cellular response to the TGFβ anti-proliferative signals and therefore decreasing cell proliferation (68).

#### Downregulated miRNA Modulating the TGFβ Pathway Activity in Thyroid Cancer

Although not downregulated in PTC samples according to TCGA (8), the downregulation miR-663 has been reported in PTC tumors when compared with normal adjacent tissues by real-time PCR (*n* = 91) (69). The same authors showed that miR-663 expression was reduced in extrathyroidal invasion PTC tissues compared with non-extrathyroidal invasion PTCs by microarray analysis. A subsequent analysis showed that miR-663 directly targets TGFβ1, thereby regulating the expression of EMT markers and matrix metalloproteinases to suppress PTC cell invasion and migration (70).

miR-7 and miR-144 have been described as downregulated in FTC as compared with follicular thyroid adenoma tissues, and both are reported to target TGFβRII (71). Both miRNAs are also downregulated in PTC (8, 72, 73), and they have been described to functionally act as tumor suppressor miRNAs by reducing cell aggressiveness.

**Figure 3:**
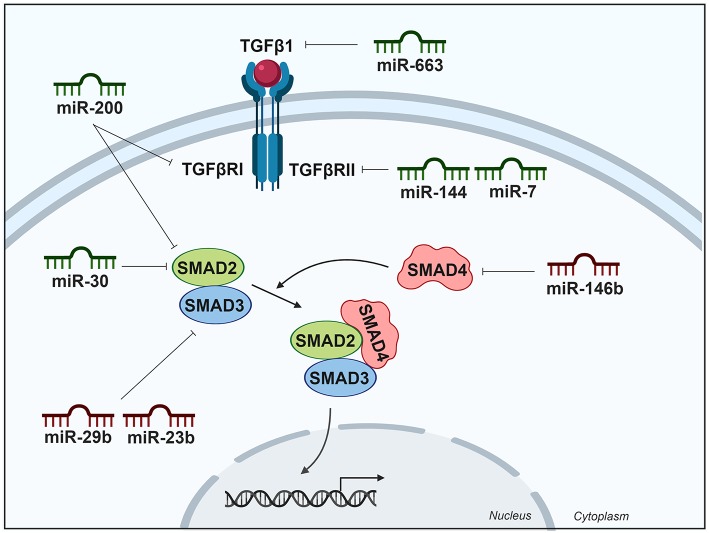
Differentially expressed miRNAs in thyroid cancer tissues modulating the TGFβ pathway. Green miRNAs represent downregulated miRNAs when comparing tumoral vs. control tissue and red miRNAs represent the upregulated. Created with BioRender.

The miR-30 and miR-200 families, which are downregulated in ATC (29, 74), have been involved in the inhibition of TGFβ signaling. The expression of these microRNAs in ATC cells reduced their invasive potential and induced mesenchymal-epithelial transition by regulating the expression of the marker proteins of this process. The miR-200 family targets TGFβRI and SMAD2, which are upregulated in most primary ATC. Moreover, miR-30 family members also target and decrease SMAD2 expression levels (29). A summary of miRNAs and their targets in the TGFβ pathway is shown in Table 1 and is schematically represented in Figure 3.

## Concluding Remarks

It is currently well-accepted that in human carcinogenesis one of the mechanisms involved is the deregulation of miRNAs through several mechanisms such as epigenetic changes, impaired transcription, amplification or deletion of miRNA genes and defects in the miRNA biogenesis machinery. In the context of thyroid cancer, several dysregulated miRNAs have been shown to affect the hallmarks of these tumors including proliferative signaling and growth suppressor evasion, or affecting invasion and metastasis.

In this review, we have described the up- and down-regulated miRNAs that target the components of the three main signaling pathways activated in thyroid cancer—MAPK, PI3K, and TGFβ. After an exhaustive review of the bibliography, we observed that both up- and down-regulated miRNAs play important roles in these pathways; specifically, that the upregulated miRNAs target factors that suppress pathway activation, whereas the downregulated miRNAs target factors activating the pathways. In this balanced fashion, the three pathways remain activated in cancer with miRNAs functioning as crucial modulators of pathway activity. Given their important roles across thyroid tumorigenesis, miRNAs can be considered as therapeutic targets, since the fine-tuning of their expression can modulate the activity of fundamental pathways hyperactivated in cancer.

## Author Contributions

JR-M performed an exhaustive revision of the bibliography. JR-M and PS wrote the manuscript.

### Conflict of Interest Statement

The authors declare that the research was conducted in the absence of any commercial or financial relationships that could be construed as a potential conflict of interest.
